# How to assess and treat right ventricular electromechanical dyssynchrony in post-repair tetralogy of Fallot: insights from imaging, invasive studies, and computational modelling

**DOI:** 10.1093/europace/euae024

**Published:** 2024-01-24

**Authors:** Miroslav Ložek, Jan Kovanda, Peter Kubuš, Michal Vrbík, Lenka Lhotská, Joost Lumens, Tammo Delhaas, Jan Janoušek

**Affiliations:** Children’s Heart Center, 2nd Faculty of Medicine, Charles University in Prague and Motol University Hospital, V Úvalu 84, 150 06 Prague, Czech Republic; Department of Biomedical Informatics, 1st Faculty of Medicine, Charles University in Prague, Kateřinská 1660/32, 121 08 Prague, Czech Republic; Children’s Heart Center, 2nd Faculty of Medicine, Charles University in Prague and Motol University Hospital, V Úvalu 84, 150 06 Prague, Czech Republic; Children’s Heart Center, 2nd Faculty of Medicine, Charles University in Prague and Motol University Hospital, V Úvalu 84, 150 06 Prague, Czech Republic; Children’s Heart Center, 2nd Faculty of Medicine, Charles University in Prague and Motol University Hospital, V Úvalu 84, 150 06 Prague, Czech Republic; Czech Institute of Informatics, Robotics, and Cybernetics, Czech Technical University in Prague, Jugoslávských partyzánů 1580/3, 160 00 Prague, Czech Republic; Maastricht University Medical Center, CARIM School for Cardiovascular Diseases, Universiteitssingel 50, 6200 MD Maastricht, The Netherlands; Maastricht University Medical Center, CARIM School for Cardiovascular Diseases, Universiteitssingel 50, 6200 MD Maastricht, The Netherlands; Children’s Heart Center, 2nd Faculty of Medicine, Charles University in Prague and Motol University Hospital, V Úvalu 84, 150 06 Prague, Czech Republic

**Keywords:** Tetralogy of Fallot, Right bundle branch block, Right ventricular dysfunction, Cardiac resynchronization therapy, Digital Twin

## Abstract

**Background and Aims:**

Right bundle branch block (RBBB) and resulting right ventricular (RV) electromechanical discoordination are thought to play a role in the disease process of subpulmonary RV dysfunction that frequently occur post-repair tetralogy of Fallot (ToF). We sought to describe this disease entity, the role of pulmonary re-valvulation, and the potential added value of RV cardiac resynchronization therapy (RV-CRT).

**Methods:**

Two patients with repaired ToF, complete RBBB, pulmonary regurgitation, and significantly decreased RV function underwent echocardiography, cardiac magnetic resonance, and an invasive study to evaluate the potential for RV-CRT as part of the management strategy. The data were used to personalize the CircAdapt model of the human heart and circulation. Resulting Digital Twins were analysed to quantify the relative effects of RV pressure and volume overload and to predict the effect of RV-CRT.

**Results:**

Echocardiography showed components of a classic RV dyssynchrony pattern which could be reversed by RV-CRT during invasive study and resulted in acute improvement in RV systolic function. The Digital Twins confirmed a contribution of electromechanical RV dyssynchrony to RV dysfunction and suggested improvement of RV contraction efficiency after RV-CRT. The one patient who underwent successful permanent RV-CRT as part of the pulmonary re-valvulation procedure carried improvements that were in line with the predictions based on his Digital Twin.

**Conclusion:**

An integrative diagnostic approach to RV dysfunction, including the construction of Digital Twins may help to identify candidates for RV-CRT as part of the lifetime management of ToF and similar congenital heart lesions.

**What’s new?**

- Right ventricular (RV) mechanical dyssynchrony caused by right bundle branch block (RBBB) displays components of a classic dyssynchrony pattern well known from left ventricular dyssynchronopathy.
- Right ventricular cardiac resynchronization therapy (RV-CRT) is able to reverse this pattern resulting in acute improvement of RV function.
- Biomedical modelling and the construction of Digital Twins may be helpful in quantifying relative impact of RV pressure and volume overload and dyssynchrony on RV function and in selecting proper RV-CRT candidates.
- Such approach, along with correction of RV pressure and volume overload, may be beneficial in the lifetime management of tetralogy of Fallot and similar congenital heart lesions.

## Introduction

Subpulmonary right ventricular (RV) dysfunction and failure are associated with a number of congenital heart lesions, typically post-repair tetralogy of Fallot (ToF).^1^ They are attributed to several factors including myocardial fibrosis, surgical scar, and long-term post-repair volume overload caused by pulmonary regurgitation. Right bundle branch block (RBBB) and resulting RV electromechanical discoordination is the most common dyssynchrony pattern in post-repair congenital heart disease and is thought to play an important role in the disease process.^2^ Pulmonary re-valvulation is thought to reverse pathologic RV remodelling. However, a decreased probability of reverse remodelling after pulmonary re-valvulation has been reported in patients with high RV volumes, low ejection fraction, and wide QRS complex (≥160 ms) due to RBBB.^3^ Limited data suggest a positive impact of RV-CRT on both acute and long-term haemodynamics in surgically repaired congenital heart disease with RV dysfunction and RBBB.^4,5,6,7,8^ Long-term efficacy and its role in life-long patient management is, however, unknown. Studies in adult patients undergoing CRT for dyssynchronous left ventricular ischaemic or idiopathic dilated cardiomyopathy have clearly shown an alignment between successful electromechanical resynchronization, acute haemodynamic effect, and long-term CRT benefit.^9^ A similar association might be implied for a dyssynchronous pulmonary RV. However, not only are specific indication criteria lacking, but implantation of a CRT system also carries potential long-term risks and drawbacks associated with the pacing hardware, specifically infection and CMR incompatibility in case of epicardial systems. Thus, prediction of relative effects of pulmonary re-valvulation and RV-CRT would be of importance. The aim of the study was to present an insight into mechanical consequences of RBBB in congenital heart disease and to describe potential use of the construction of Digital Twins in the disease management.

## Methods

Two patients with repaired ToF and significantly decreased RV function (Patients #1 and #2; *Table 1*) were evaluated. Both had complete RBBB, significant pulmonary regurgitation, and decreased exercise tolerance, while Patient #2 had also a pulmonary valve stenosis. Patient #1 underwent a Melody valve implantation and Patient #2 surgical RV to pulmonary artery conduit implantation with placement of epicardial leads for permanent RV-CRT.^7^ Echocardiography including speckle tracking imaging and CMR or cardiac CT scan was performed prior to and 8 and 10 months after re-intervention, respectively. The acute effects of RV-CRT were studied during a clinically indicated cardiac catheterization procedure prior to the re-interventions.

**Table 1:** Clinical and imaging data

Parameter	Patient #1		Patient #2	
	Baseline	PVR^a^	Baseline	PVR^b^ + RV-CRT
Clinical data				
Age (years)	16.5	17.6	10.9	12.6
Sex	Male		Male	
Weight (kg)	59.5	62.4	46.6	60.0
Time from intervention (months)	—	7.5	—	9.9
QRS duration (ms)	193	190	169	110
NYHA class	1	1	2	1
Maximum workload (W/kg body weight)	3.0	3.0	2.1	3.0
% Predicted	71.4	73.2	47.7	68.2
Maximum oxygen uptake (mL/kg body weight/min)	33.1	28.3	25.0	37.5
% Predicted	64.9	56.4	53.0	79.4
SD from normal population	−3.7 SD	−3. 9 SD	−4.8 SD	−2.1 SD
NTproBNP (normal upper limit) (ng/mL)	179 (157)	79.4 (125)	293 (157)	<5.0 (157)
Echocardiography				
Pulmonary stenosis peak/mean (mmHg)	0	15/18	55/30	0
Pulmonary regurgitation (grade)	Severe	None	Severe	Mild
Tricuspid regurgitation (grade)	Moderate	Moderate	Mild	None
RV + dP/dt (mmHg/s) from tricuspid regurgitation	318	197	463	—
RV myocardial performance index	0.08	0.16	0.36	0.24
RV septal-to-lateral mechanical delay (ms)	128	138	130	2
RV systolic stretch fraction	0.42	0.43	0.51	0.11
RV wasted work ratio	0.32	NA	0.25	NA
Cardiac magnetic resonance/cardiac CT scan				
RV end-diastolic volume index (mL/sqm BSA)	182	151	188	128
RV ejection fraction (%)	36	37	38	43
Pulmonary regurgitant fraction (%)	23	1	42	—
LV end-diastolic volume index (mL/sqm BSA)	89	90	84	96
LV ejection fraction (%)	50	48	57	64

BPM, beats per minute; BSA, body surface area; CT, computed tomography; LV, left ventricular; NA, not available; NYHA, New York Heart Association; PVR, pulmonary re-valvulation; RV, right ventricular; RV-CRT, RV cardiac resynchronization therapy; SD, standard deviation; sqm, square metre.

^a^Percutaneous Melody valve implantation.

^b^Right ventricular to pulmonary artery conduit.

Echocardiographic measurements were described in detail in our previous publication.^5^ In brief, pulmonary and tricuspid regurgitation was graded as none, mild, moderate, or severe. Speckle tracking echocardiography derived longitudinal segmental strain in the apical four-chamber view was used to assess the components of classic pattern RV dyssynchrony^10^ and to calculate maximum systolic RV septal-to-lateral mechanical delay, RV systolic stretch fraction, and RV wasted work ratio. Right ventricular systolic stretch fraction reflects the ratio of total stretch over total shortening during systole. The wasted RV work ratio was calculated in accordance with a published report.^11^ Higher numbers indicate decreased RV global contractile efficiency.

The effect of acute RV-CRT was tested in both patients at baseline during catheterization by means of atrial triggered pacing from the late activated lateral RV wall in complete fusion with the intrinsic activation as described earlier^5^ to achieve shortest possible QRS complex duration. Data were obtained to reason for potential indication for permanent RV-CRT later on.

The CircAdapt model of the human heart and circulation (www.circadapt.org), a well-validated model for simulation of myocardial mechanics and cardiovascular haemodynamics in dyssynchronous hearts,^12,13,14,15,16^ was used to create Digital Twins at baseline. To that end, ventricular volumetric data and valve characteristics obtained at baseline as well as RV septal-to-lateral mechanical delay derived from speckle tracking echocardiography using longitudinal strain in the apical four-chamber view (*Table 1*) were used to create the initial models for both patients. The CircAdapt model has been shown to be very versatile in representing ventricular activation patterns.^17^ Gradual prolongation of the RV mechanical delay was programmed from the septum along both the anterior and inferior walls towards the RV free wall in six segments (inferoseptal, anteroseptal, anterior, anterolateral, inferolateral, inferior) from 0 ms in the inferoseptal segment to the maximum delay in the anterolateral segment to accomplish the reported activation pattern in repaired ToF.^18^ This method has previously been shown to produce realistic simulations of regional LV and RV stresses and strains as well as system-level haemodynamics in the dyssynchronously activated heart,^19,20^ as validated using both clinical and experimental data.

In the thus-created Digital Twins, we simulated pulmonary re-valvulation, RV-CRT, and their combination. For the purpose of RV-CRT simulation, the RV septal-to-lateral mechanical delay was set to zero. The modelling results reflect acute haemodynamic changes caused by each of the interventions. Right ventricular and LV pressure–volume and myofibre stress–strain curves, quantitative estimates of ventricular contractility, pump work, and myocardial work were calculated. Right ventricular and LV myocardial work were both calculated as the work of their respective free wall + (septal work/2) to compensate for the fact that the septum participates in both the RV and LV contraction and relaxation. Right ventricular myocardial work efficiency was calculated as RV myocardial work/RV pump work. Right ventricular systolic stretch fraction and RV wasted work ratio were calculated from modelled segmental strain curves in analogy to calculations from speckle tracking imaging. Influence of the interventions on exercise capacity was modelled according to Lumens *et al.*^21^ Additionally, the impact of RV-CRT on myocardial work efficiency as well as maximum cardiac output during exercise was modelled as a function of different degrees of pulmonary stenosis or regurgitation using baseline patient data.

Ethical approval was not required by the institutional ethical board because of purely retrospective data acquisition and anonymized presentation without any impact on clinical patient management. However, informed consent from the legal guardians was obtained for the clinically indicated invasive study including assessment of RV dyssynchrony as well as for all interventions applied.

All data used in this manuscript are available at the authors upon reasonable request.

## Results

Clinical and imaging data quantifying RV function and dyssynchrony caused by RBBB at baseline and after intervention are summarized in *Table 1*. At baseline, several, but not all, components of classic pattern dyssynchrony including early septal contraction and lateral RV wall stretching followed by late lateral wall contraction peaking after pulmonary valve closure were present. Right ventricular septal-to-lateral mechanical delay was associated with wasted RV free wall shortening that extends beyond RV ejection, coincides with peak negative RV free wall longitudinal strain, and causes a septal flash visible as a late systolic left-to-right septal shift (Graphical abstract; data obtained from Patient #2).

**Figure 1:**
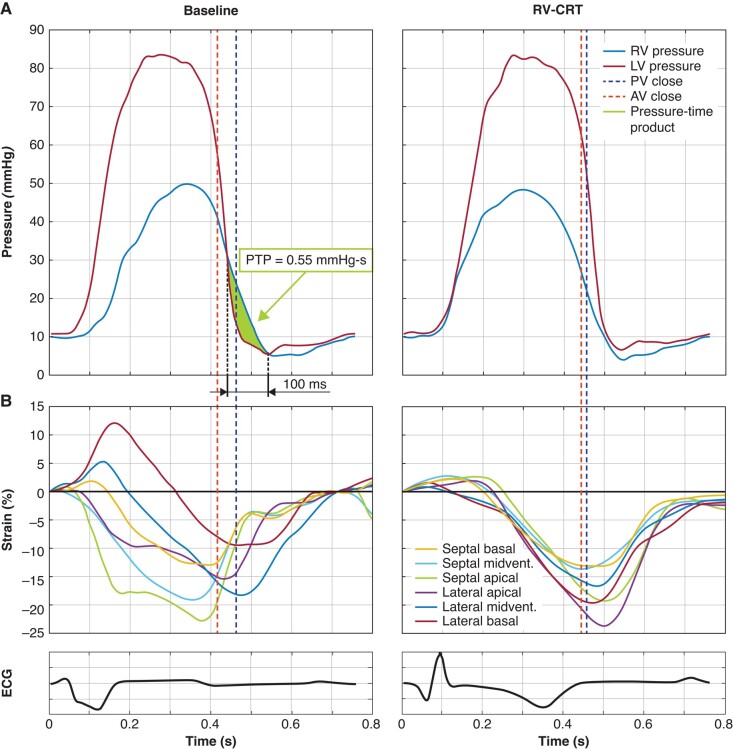
Measured data from Patient #2 at invasive study during baseline condition and after RV-CRT. (*A*) Right ventricular and LV pressure curves from the invasive study. The timespan during end-systole at baseline in which RV pressure exceeds LV pressure (left) is abolished after RV-CRT (right). The PTP area depicts the related pressure–time product (PTP) (see *Table 1* for respective data). (*B*) Right ventricular longitudinal segmental strain curves showing components of a classic dyssynchrony pattern at baseline with early basal, midventricular and apical septal contraction with accompanying stretch of the RV free wall (lateral basal and midventricular) segments followed by late contraction of the latter. During RV-CRT, all RV segments are contracting synchronously. AV close, aortic valve closure; LV, left ventricle; PV close, pulmonary valve closure; PTP, pressure–time product; RV, right ventricle; RV-CRT, RV cardiac resynchronization therapy.

Right ventricular mechanical discoordination due to RBBB was correctable by acute RV-CRT performed during the invasive study. Right ventricular cardiac resynchronization therapy resulted in immediate disappearance of septal flash (see Supplementary material online, *Movie clip 1*) and acute improvement of RV function and mechanics as confirmed by increase in RV contractility and elimination of delayed RV contraction (*Table 2*, *Figure 1A*). Speckle tracking echocardiography confirmed immediate elimination of RV contractile discoordination by RV-CRT (*Figure 1B*).

**Table 2:** Effects of acute RV-CRT measured during invasive study

Parameter	Patient #1		Patient #2	
	RV-CRT off	RV-CRT on	RV-CRT off	RV-CRT on
Heart rate (BPM)	65	65	60	60
QRS duration (ms)	193	95	169	96
RV + dP/dt (mmHg/s)	289	470	411	456
Onset QRS to peak RV + dP/dt (ms)	125	107	155	83
LV + dP/dt (mmHg/s)	659	670	775	662
Onset QRS to peak LV + dP/dt (ms)	117	128	117	119
Time RV pressure exceeding LV pressure in end-systole (ms)	55	37	100	0
Pressure–time product of RV pressure exceeding LV pressure in end-systole (mmHg/s)^a^	0.33	0.14	0.55	0.00

For abbreviations, see *Table 1*.

^a^See *Figure 1A* for explanation.

At 8 and 10 months after pulmonary re-valvulation (plus RV-CRT in Patient #2), both patients showed decreased RV size accompanied by mild increase in RV ejection fraction in Patient #2 who also showed improved exercise tolerance (*Table 1*). Also, only Patient #2 showed abolishment of septal-to-lateral RV mechanical dyssynchrony and increased RV contraction efficiency, presumably as a consequence of RV-CRT, an effect already reported in a previous study.^5^

**Figure 2:**
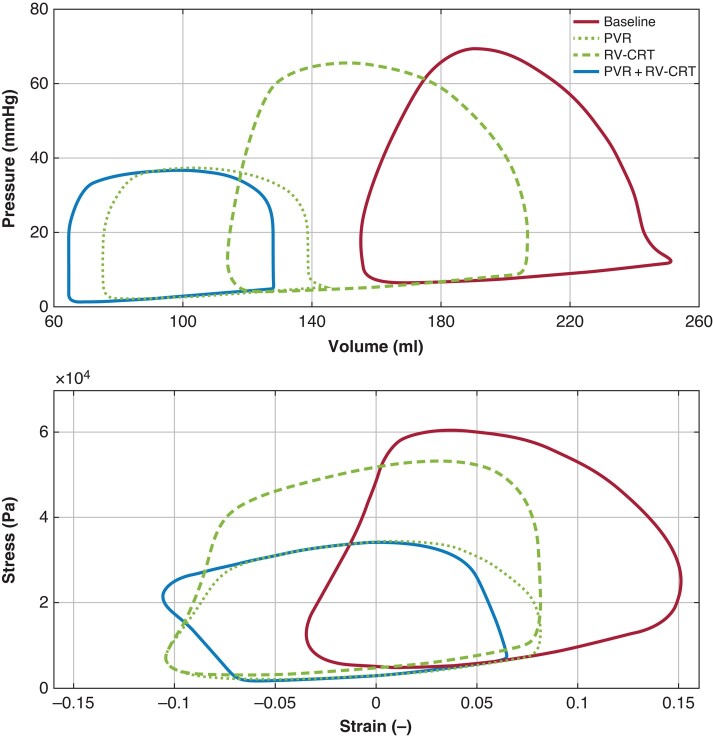
Modelled data from Patient #2. The pressure–volume and strain–stress loops reflect the combination of RV volume load and dyssynchrony at baseline with major leftward shift of the loops after volume unloading and further change after RV-CRT. PVR, pulmonary valve replacement; RV-CRT, RV cardiac resynchronization therapy.

**Figure 3:**
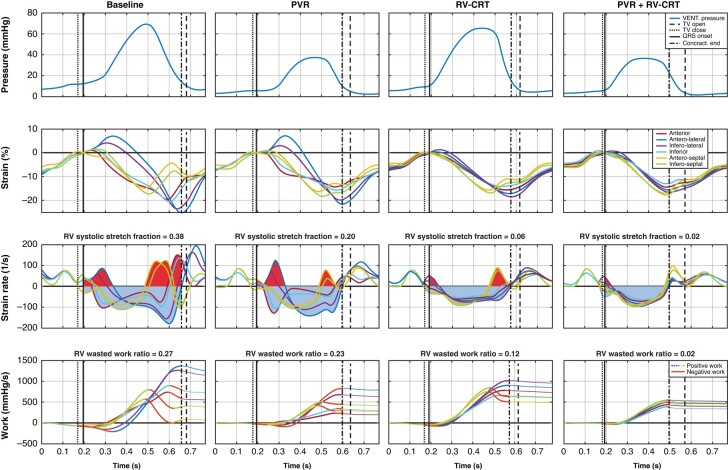
Modelled data from Patient #2 at baseline and after pulmonary re-valvulation, RV-CRT, and their combination. From top to bottom: (i) RV pressure curve. (ii) Modelled segmental strain curves displaying circumferential RV strain in individual RV segments and showing late mechanical activation of the RV free wall at baseline with major improvement of segmental shortening coordination after RV-CRT. (iii) Modelled calculation of systolic stretch fraction. Systole is defined as the time from QRS onset to latest peak negative strain (contraction end). The negative area under respective strain rate curves reflects systolic segmental shortening; the positive area corresponds with systolic segmental stretch. Paradoxical RV stretch during systole is greatly diminished after RV-CRT resulting in significant decrease in RV systolic stretch fraction. (iv) Modelled calculation of wasted work ratio using work performed by individual segments. Desceding parts of the segmental curves reflect negative work during systole and are displayed in red. Major decrease in RV wasted work ratio is seen after RV-CRT. LV, left ventricle; PTP, pressure–time product; RV, right ventricle; RV-CRT; TV close, tricuspid valve closure; TV open, tricuspid valve opening. For other abbreviations, see *Figure 2*.

The Digital Twins (*Table 3*) showed a decrease in both the RV pump work and myocardial work after pulmonary re-valvulation due to alleviation of RV volume overload as reflected also by major change in the pressure–volume and stress–strain curves (*Figure 2*). Distribution of segmental myocardial work density in the RV free wall and septum remains, however, greatly inhomogeneous. Right ventricular cardiac resynchronization therapy both alone and as added to pulmonary re-valvulation led to homogenization of segmental myocardial work density with a significant decrease in the originally late contracting RV free wall and a concomitant increase in myocardial work density in the LV free wall. The modelled wasted RV work ratio and RV systolic stretch fraction^5,11^ was greatly diminished by RV-CRT indicating an improvement in RV contraction efficiency (*Figure 3*). Such improvement was also accompanied by decrease in RV myocardial work expedited per 1 L/min of cardiac output and by a decrease of the myocardial-to-pump work ratio—an effect not seen after modelled isolated relief of RV volume overload.

**Table 3:** Computational modelling of pulmonary re-valvulation, right ventricular cardiac resynchronization therapy, and their combination

Parameter	Modelled patient #1				Modelled patient #2			
	Baseline	PVR^a^	RV-CRT	PVR + RV-CRT	Baseline	PVR^b^	RV-CRT	PVR + RV-CRT
RV + dP/dt (mmHg/s)	157	171	247	243	363	279	431	345
LV + dP/dt (mmHg/s)	606	604	585	605	656	668	697	719
RV pump work (J/cycle)	0.36	0.29	0.40	0.29	0.55	0.26	0.62	0.27
LV pump work (J/cycle)	0.61	0.63	0.64	0.65	0.61	0.62	0.62	0.61
RV myocardial work (J/cycle)	0.62	0.56	0.49	0.39	0.97	0.46	0.71	0.37
LV myocardial work (J/cycle)	0.37	0.38	0.55	0.56	0.21	0.43	0.54	0.52
Myocardial work density: RV free wall (J/m^3^/cycle)	6316	5489	4812	3477	10 438	4011	7544	3516
Myocardial work density: septum (J/m^3^/cycle)	2904	3456	3449	3685	3268	6074	3698	3890
Myocardial work density: LV free wall (J/m^3^/cycle)	2383	2372	3691	3681	1498	3174	4699	4479
Standard deviation of segmental RV myocardial work density (J/m^3^/cycle)	1849	1418	643	98	5171	2640	1813	176
RV myocardial work/cardiac output (J/L)	7.96	7.19	6.29	4.94	14.86	7.01	10.86	5.70
RV myocardial work/RV pump work	1.72	1.93	1.23	1.33	1.75	1.75	1.14	1.37
RV systolic stretch fraction	0.43	0.37	0.10	0.08	0.38	0.20	0.06	0.02
RV wasted work ratio	0.42	0.42	0.22	0.22	0.27	0.23	0.12	0.02
Modelled exercise: cardiac output at central venous pressure = 25 mmHg (L/min)	8.9	9.5	12.9	13.7	8.3	10.9	10.3	15.2

For abbreviations, see *Table 1*.

PVR, pulmonary re-valvulation; RV-CRT, right ventricular cardiac resynchronization therapy.

^a^Percutaneous Melody valve implantation.

^b^Right ventricular to pulmonary artery conduit.

**Figure 4:**
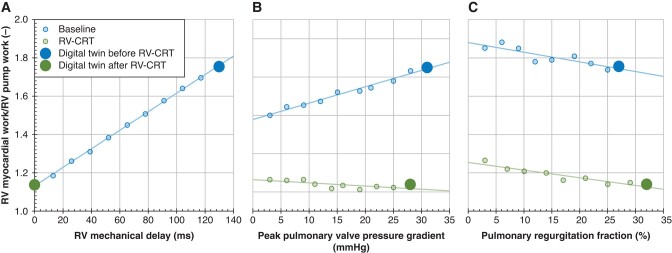
Modelled data from *Patient #2*. The relationship between RV myocardial work and RV pump work is modelled at baseline and after RV-CRT as a function of (*A*) RV mechanical delay. (*B*) Pulmonary stenosis. (*C*) Pulmonary regurgitation. RV-CRT, RV cardiac resynchronization therapy.

**Figure 5:**
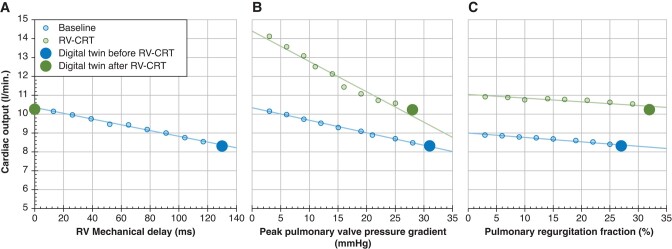
Modelled data from Patient #2. Cardiac output at maximum exercise is displayed at baseline and after RV-CRT as a function of (*A*). RV mechanical delay. (*B*) Pulmonary stenosis. (*C*) Pulmonary regurgitation. RV-CRT, RV cardiac resynchronization therapy.

Another effect of RV-CRT was that modelled exercise capacity increased to a greater degree than by pulmonary re-valvulation alone. *Figure 4A* displays decreasing RV myocardial work efficiency as reflected by RV myocardial work/RV pump work ratio with the increase in RV mechanical delay using the Digital Twin of Patient #2. It shows that RV myocardial work efficiency proportionally increased with the amount of resynchronization. In *Figure 4B*, the effect of increase of the pulmonary valve effective orifice area and, hence, decrease in pulmonary stenosis alone (blue circles) or in combination with CRT (green circles) are shown, clearly indicating that positive effect of CRT on RV myocardial work efficiency outperforms the influence of pulmonary stenosis relief. *Figure 4C* shows that decrease of pulmonary regurgitation severity decreased RV myocardial work efficiency, both in the dyssynchronous (open circles) and resynchronized RV (solid circles), though the resynchronized RV had a clearly higher RV myocardial work efficiency. *Figure 5* shows predicted exercise capacity for Patient #2 as a function of amount of dyssynchrony (*Figure 5A*) and relief of pulmonary stenosis (*Figure 5B*) or pulmonary regurgitation (*Figure 5C*) with (green circles) or without RV-CRT (blue circles). Cardiac outputs are shown for the situation in which mean pressure in the vena cava increased to 25 mmHg.^21^ It clearly shows that predicted exercise capacity increases proportionally to the amount of relief of either dyssynchrony, pulmonary stenosis, or pulmonary regurgitation (blue circles). Exercise capacity increased the most if any valvular intervention was accompanied by CRT (green circles). The modelled parameters (*Table 3*) where well in line with the clinically observed intervention effects as displayed in *Tables 1* and *2*. The semi-quantitative magnitude of clinical changes and their computational predictions after performed interventions for those parameters allowing direct comparison is displayed in *Table 4*.

**Table 4:** Comparison of clinical effects and computational modelling predictions

	Patient #1				Patient #2			
	RV-CRT		PVR		RV-CRT		PVR + RV-CRT	
Parameter	Clinical	Modelled	Clinical	Modelled	Clinical	Modelled	Clinical	Modelled
RV + dP/dt	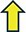	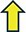	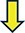	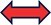	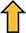	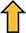	NA	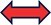
RV systolic stretch fraction	NA	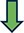	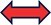	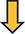	NA	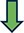	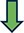	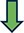
RV wasted work ratio	NA	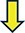	NA	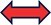	NA	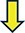	NA	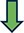
Exercise capacity	NA	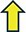	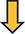	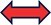	NA	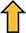	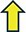	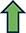
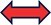	Proportional increase/decrease by 0–10%							
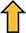 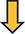	Proportional increase/decrease by 11–33%							
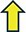 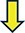	Proportional increase/decrease by 34–66%							
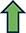 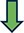	Proportional increase/decrease by 67–100%							

PVR, pulmonary re-valvulation; RV-CRT, right ventricular cardiac resynchronization therapy.

## Discussion

Standard treatment of the dysfunctional right ventricle in post-repair congenital heart disease nowadays includes pulmonary re-valvulation to relieve RV volume/pressure overload due to pulmonary regurgitation/stenosis.^1^ Right ventricular dyssynchronopathy, the other potential component of RV dysfunction,^2^ is largely not addressed, and RV-CRT to date has not been included into guideline-driven therapy. The reason lies mainly in the unclear role of RV-CRT in the lifetime patient management, the technical complexity of pacemaker implantation, and the lack of availability of magnetic resonance imaging–compatible epicardial pacing systems. Understanding of the pathophysiology of RV mechanical discoordination, its impact on contractile efficiency and myocardial energy expenditure, as well as its role in chronic pathologic RV remodelling and recognizing the potential of RV-CRT is thus of importance. Prediction of the relative effects of RV re-valvulation and RV-CRT is highly desired to improve therapy planning, the more so since RV-CRT may be easily undertaken during surgical pulmonary valve replacement if predicted to be beneficial.

Using imaging, invasive haemodynamic data, and patient-specific modelling, this study has clearly shown multiple aspects of RV mechanical dyssynchrony and the potential of RV resynchronization to improve RV mechanics, contraction efficiency, and patient’s functional capacity. Components of classic pattern RV dyssynchrony, resembling a similar picture as within the left ventricle in the presence of left bundle branch block,^10^ seem to be uniformly present and are abolished by RV-CRT. Patient-specific modelling could predict both the effect of pulmonary re-valvulation and RV-CRT, and these effects were in line with the clinically observed haemodynamic changes. Interestingly, the modelled positive effect of RV-CRT was present over a wide range of both pulmonary regurgitation severity and pulmonary stenosis gradients presenting a separate therapeutic option apart from pulmonary re-valvulation. In accordance with previously published data,^21^ we also show that RV-CRT may exert greater beneficial effect on exercise capacity than RV volume unloading. In addition, computational modelling may shed light on optimizing pacing therapies for CRT including the simulation of different pacing sites on CRT response.^22^ Thus, patient-specific modelling may lead us to the doorstep of personalized medicine in congenital heart disease by creating the patient’s individualized Digital Twin allowing to test the effect of various therapeutic interventions. The clinical outcome in Patient #2 is a result of a combined haemodynamic and CRT intervention. The digital model helps to discern these two components and may assist clinical decision-making. Recent studies^14,23^ demonstrated the ability of the CircAdapt model to reliably simulate regional RV deformation abnormalities and even to estimate regional myocardial tissue properties from regional ventricular strain patterns of individual patients with RV electromechanical disease. Nevertheless, further studies on larger patient populations are needed to verify the predictive accuracy of virtual interventions in personalized biophysical models in the context of repaired ToF .

### Limitations

The digital model does not completely match the clinical patient parameters. Thus, modelling of the changes in ventricular mechanics and contraction efficiency following simulated interventions is an approximation, showing rather trends than exact absolute values. Also, the digital model of RV-CRT implies a septal-to-lateral RV mechanical delay of zero. This is an oversimplification as RV electromechanical activation will not be totally homogenous during RV-CRT. Thus, RV-CRT effects may be overestimated by the model. Further, in the clinical setting, exercise capacity was measured using oxygen uptake, whereas cardiac output was predicted by modelling. Although both variables have a linear correlation with similar proportional increments, direct comparison of maximum values before and after intervention is possible just under the presumption of identical arterio-venous oxygen difference and maximum heart rate achieved, respectively. While the first could not be measured, the second has been fulfilled in both patients. Additionally, the model cannot well account for haemodynamic changes induced by reverse remodelling after interventions were performed and reflects rather acute changes caused by removal of RV pressure/volume overload and RV dyssynchrony, respectively. Although creating Digital Twins for a higher number of patients would potentially strengthen the results, we do believe that the two patients described reflect well the spectrum of post-repair ToF with different degrees of pulmonary regurgitation and stenosis and give valuable insights into the disease pathophysiology.

In conclusion, RBBB causes significant RV mechanical discoordination and inefficiency with late systolic right-to-left septal shift. This leftward septal flash, an easily visible signature of RV dyssynchrony, is caused by RV free wall contraction being delayed relative to LV free wall and septal contraction. Right ventricular cardiac resynchronization therapy corrects the mechanical derangement and improves multiple haemodynamic indices in congenital heart disease patients with RV electromechanical dyssynchrony. In the future, patient-specific modelling may help to describe RV-CRT effects and to predict RV-CRT efficacy at individual patient level.

## Supplementary Material

euae024_Supplementary_Data

## Data Availability

All data used in this study and not provided within the manuscript are available at the authors upon reasonable request.
